# Analgesic and anti-inflammatory activities of *Vitex trifolia* extract and development of a topical herbal cream

**DOI:** 10.1016/j.jaim.2026.101393

**Published:** 2026-09-08

**Authors:** Wanhuda Paduka, Nongluk Kunworarath, Kanchapat Mahaprom, Jomkarn Naphatthalung, Julalak Chokpaisarn

**Affiliations:** a Traditional Thai Medical Research and Innovation Center, Faculty of Traditional Thai Medicine, Prince of Songkla University, Hat Yai, Songkhla, 90110, Thailand; b Traditional Thai Medicine Hospital, Faculty of Traditional Thai Medicine, Prince of Songkla University, Hat Yai, Songkhla, 90110, Thailand

**Keywords:** *Vitex trifolia* L., Anti-inflammatory activity, Herbal cream product, Skin disorders

## Abstract

**Background:**

*Vitex trifolia* L. is a medicinal plant traditionally used in Thai medicine for treatment of muscle pain and skin disorders.

**Objective:**

To evaluate the *in vivo* analgesic and anti-inflammatory activities of the ethanolic extract of *Vitex trifolia* (VTE) and to develop a stable topical herbal cream.

**Materials and methods:**

The analgesic activity of VTE was evaluated using acetic acid-induced writhing and hot plate tests. The anti-inflammatory activity was assessed using ethyl phenylpropiolate (EPP)-induced ear edema and carrageenan-induced paw edema models in rats. A topical cream was developed containing VTE at 0.4% w/w, and its physical characteristics (pH, viscosity, spreadability, and phase separation), short-term and accelerated stability (at 4 °C and 30 °C for 90 days), and phytochemical contents (phenolics, flavonoids, and proanthocyanidins) were analyzed.

**Results:**

VTE (0.5-2.0 mg/ear) significantly reduced EPP-induced ear edema by up to 71.5% at 120 min (p < 0.001) and suppressed carrageenan-induced paw edema by 45.3% at 4 h with a 250 mg/kg body weight (p < 0.001), showing comparable efficacy to standard drugs. The number of abdominal writhes in mice treated with VTE remained statistically comparable to the control group (p > 0.05), and no significant increase in latency time was observed in the hot plate model. The HCF5 cream exhibited favorable physical characteristics (pH: 4.26-4.99; viscosity: 8683–40,065 cP), and remained stable over 90 days, especially at 30 °C. Phytochemical analysis showed stable retention of total phenolics, flavonoids, and proanthocyanidins with no significant degradation during storage.

**Conclusion:**

This study confirms the anti-inflammatory potential of VTE and its suitability for use in a stable topical herbal formulation.

## 1 Introduction

Inflammation is a fundamental response of the human body which stimulated by several factors such as physical injury, microbial infection, or chemical irritants [1]. However, uncontrolled inflammation can cause various pathological conditions, including dermatitis, eczema, as well as musculoskeletal disorders [1]. Currently, conventional anti-inflammatory drugs, particularly non-steroidal anti-inflammatory drugs (NSAIDs), are effective for inflammatory treatment. Nevertheless, prolonged use may lead to several side effects, including skin irritation as well as skin hypersensitivity [2]. To address these concerns, the development of safe and effective topical formulations from herbs has gained growing attention in recent years.

*Vitex trifolia* L. subsp. litoralis Steenis (Lamiaceae) is a medicinal plant distributed widely across tropical and subtropical regions of Asia. In ethnomedicine, particularly in the Southern part of Thailand, this plant has been extensively utilized for treatment of inflammatory conditions, especially skin disorders [3]. Our previous work revealed that *V. trifolia* possessed potent free radicals scavenging activity, promoted cell migration, and inhibited the growth of skin-associated bacterial pathogens without exhibiting cytotoxicity [4]. Moreover, the phytochemical investigation displayed that *V. trifolia* contains several bioactive compounds, such as apigenin 7-*O*-glucoside, caffeic acid, α-mangostin, luteolin, as well as casticin [4]. These compounds are commonly known as an antioxidant, anti-inflammatory, and antimicrobial agents [[4], [5], [6], [7]].

Topical delivery systems are frequently considered the preferred route for managing localized inflammatory conditions [8,9]. Recent research efforts have focused on developing topical herbal products with optimized physicochemical properties, stability, and release profiles to ensure their therapeutic efficacy [[10], [11], [12], [13]]. In this study, the development of a stable topical formulation containing *V. trifolia* presented a promising strategy to harness the therapeutic benefits of this plant in a modern dosage form. Even, there were several scientific studies on *in vitro* bioactivities. However, there remains a significant gap in the scientific literature regarding its *in vivo* pharmacological efficacy and formulation stability, particularly in the dosage form of topical applications. Therefore, the present study aims to evaluate the analgesic and anti-inflammatory effects of ethanolic extract of *V. trifolia* (VTE) using *in vivo* animal models. Furthermore, the development and stability of a VTE herbal cream were investigated. By integrating ethnopharmacological knowledge with experimental validation, this research seeks to substantiate the traditional uses of *V. trifolia* and contribute to the advancement of effective therapies for managing pain, inflammation, and skin conditions in healthcare system.

## 2 Methods

### 2.1 Chemicals and drugs

Acetic acid (CH_3_COOH) (RCI Labscan®), Carrageenan (Sigma Aldrich, Singapore), Morphine sulfate injection (Food and drug administration, Nonthaburi Province, Thailand), Indomethacin (Sigma Aldrich, Singapore), Ethyl phenylpropiolate (Sigma Aldrich, Singapore), Acetone (Grade AR), Thiopental sodium sterile (Pharmacia limit), Ethanol, Propylene glycol (Cosmetic grade), Tween 80 (Cosmetic grade), and Distilled water.

### 2.2 Plant material and extract preparation

Fresh leaves of *Vitex trifolia* L. were collected from Songkhla Province, Southern Thailand, and authenticated by a botanist at the Faculty of Traditional Thai Medicine, Prince of Songkla University. A voucher specimen (VT2023-01) was deposited in the department herbarium. The leaves were washed, shade-dried, and ground into a coarse powder. Extraction was performed using 95% ethanol by maceration at room temperature for 14 days with intermittent agitation. The extract was filtered by a Buchner suction filter and concentrated in a rotavapor under reduced pressure and controlled temperature (50-60 °C) to give an extract with a 17.07 % percentage yield, which was kept at −21 °C until further use.

### 2.3 Animals

The animals (ICR mice of 25-40 g and Wister rats of 150-300 g) were purchased from Nomura Siam International Co., Ltd., Pathumwan, Bangkok. Only male animals were used to minimize variability due to hormone fluctuations that may affect inflammation and pain perception. Animals were brought to stay at the Lab Animals Service Center, Faculty of Science, Prince of Songkla University, Hat Yai Campus, for 1 week before the experiment. They were kept in clean polypropylene cages with aspen wood chip (replaced every three days) under controlled temperature (21 ± 3 °C), humidity remains 50 ± 10%, maintaining a 12/12 h light/dark cycle. Standard pellets as basal diet and water ad libitum were supplied to the animals for one week. After fasting overnight, the animal was weighed before the experiment. All procedures involving animals were conducted in accordance with the guidelines of the Prince of Songkla University Animal Ethics Committee. The animal ethics application was submitted on 11 November 2022 and approved on 1 December 2022, under approval number (Ethic Number: MHESI 68014/2083, Ref.AR103/2022).

### 2.4 Anti-inflammatory activity study

#### 2.4.1 Ethyl phenylpropiolate (EPP)-induced rat ear edema

The method described by Dunstan et al. (1997) and Wanikiat et al. (2008) was used with slight modification to assess the topical anti-inflammatory activity of *V. trifolia* [14,15].

Male rats of 150-300 g body weight were randomly divided into five groups of six rats each. The vehicle (Ethanol:Acetone, 4:6) 20 μl/ear, Indomethacin 2 mg/20μl/ear, and VTE (0.5, 1 and 2 mg/20μl/ear) were applied topically to the inner surfaces of rat ear using an automatic microliter pipette. After 30 min, EPP (1 mg/20μl/ear, dissolved in acetone) was applied topically to the outer surfaces of the rat ear. The thickness of each ear was measured with a pocket thickness gauge before and at 15, 30, 60, and 120 min after edema induction. The increase in ear thickness was compared with the control group, and the percent inhibition was calculated according to the following formula.

%Inhibition=Edema(control)−Edema(sample)/Edema(control)×100

#### 2.4.2 Carrageenan-induced rat paw edema

The efficacy of *V. trifolia* in reducing induced inflammation was evaluated by the acute inflammation method in rats as designated [16,17].

Male rats weighing 150-300 g were used. All animals were withdrawn from food 12 h before the start of experiment and animals were divided into five groups (n = 6): Group 1 received vehicle (Control, Propylene glycol: Tween 80: Distilled water, p.o.), Group 2 received standard drug indomethacin (10 mg/kg, p.o.), Groups 3-5 received VTE (125, 250, and 500 mg/kg, p.o.). After 30 min, all the animals of each group received 1% carrageenan (0.1 ml) into the sub-planter surface of right hind paw. The volume of the right paw was measured at 0, 1, 2, 3 and 4 h. Inhibitory activity was calculated by the following formula:

%Inhibition=Edema(control)−Edema(sample)/Edema(control)×100

### 2.5 Anti-nociceptive activity study

#### 2.5.1 Acetic acid-induced writhing test

Acetic acid-induced writhing method, as developed by Rauf et al. (2014), was used to identify peripheral anti-nociceptive activity of the plant extracts of *V. trifolia* [18].

ICR mice of male sex weighing 25-40 g were used. All animals were withdrawn from food 3 h before the start of experiment and animals were divided into five groups (n = 6): Group 1 received vehicle (Control, Propylene glycol: Tween 80: Distilled water, p.o.), Group 2 received standard drug indomethacin (10 mg/kg, p.o.), Groups 3-5 received VTE (125, 250, and 500 mg/kg, p.o.). Thirty min post-dosing, 0.6% acetic acid (10 ml/kg) was injected intraperitoneally. The number of cumulative writhes was documented for 30 min. Inhibition percentage was calculated as follows:

$$\%\;\text{Inhibition}=\left(A_{\text{control}}\;–\;B_{\text{test}}\right)/A_{\text{Control}}\times 100$$

A_control_ is the mean writhes in the control group and B_test_ is the mean writhes in the test groups.

#### 2.5.2 Hot plate test

Hot plate method, as developed by Bannon & Malmberg. (2007), and Siddiqui et al. (2018) was used to identify the central anti-nociceptive activity of the plant extracts of *V. trifolia* [16,19].

ICR mice of male weighing 25-40 g were used. All animals were withdrawn from food 3 h before the start of experiment. The mice that showed responses to the hot plate thermal stimulation after 4 s were selected for the test. In this case, the hot plate temperature was kept at 55 ± 1.0 °C, and the period taken to show the response by hind paw licking or jumping was documented in seconds. Animals were divided into five groups (n = 6): Group 1 received vehicle (Control, Propylene glycol: Tween 80: Distilled water, p.o.), Group 2 received standard drug morphine (10 mg/kg, injected subcutaneously; s.c.), Groups 3-5 received VTE (125, 250, and 500 mg/kg, p.o.). Morphine was used as the reference drug in this experiment because it is a well-established centrally acting analgesic, widely used as a positive control in thermal nociception models. The dose of 10 mg/kg administered subcutaneously is commonly reported to produce a consistent and robust antinociceptive response in rodents [16]. Each mouse was then placed on the plate, and the latency of mice to the thermal stimulus was recorded at 0, 30, 60, 90, and 120 min post-treatment. Response time was recorded when the animal first licked its paws or started to jump. To avoid mouse tissue damage, 30 s was chosen as the cut-off time.

### 2.6 *V. trifolia* pharmaceutical formulation as an herbal cream

For herbal cream formulation, five *V. trifolia* cream formulations were prepared using several ingredients listed in Table 1. Multiple emulsifiers were incorporated into the cream formulations to improve emulsion stability, texture, viscosity, and spreadability of the topical product. The combination of emulsifiers with different hydrophilic–lipophilic balance properties was intended to enhance the stability of the oil-in-water emulsion system and minimize phase separation during storage [20]. Variations in emulsifier and preservative concentrations among formulations were designed to optimize physicochemical stability, microbial protection, and overall formulation performance, thereby identifying the most suitable formulation for topical application [20]. For the pharmaceutical preparation, the oil phase, which consisted of the emulsifier and other oil-soluble components, was heated to 45 °C in a water bath, while the water-soluble components were added to water and heated, followed by the addition of the extract. Then, the oil phase was incorporated into the water phase under continuous stirring until the emulsion cooled. The concentration of the VTE was maintained at 0.4% w/w based on its significant *in vivo* anti-inflammatory activity. Physical characteristics of all formulations, such as color, odor, consistency, phase separation, pH, and viscosity were determined [21].

**Table 1:** The ingredients of five formulations of herbal *V. trifolia* cream formulation (HCF).

Components	Formulations (%w/w)					Functions
	HCF1	HCF2	HCF3	HCF4	HCF5	
VTE	0.4	0.4	0.4	0.4	0.4	Active ingredients
Liquid paraffin	10	10	10	5	5	Emollients
White soft paraffin	10	10	10	10	10	Emollients
Cetyl alcohol	10	10	10	6	3	Stiffening agent/Thickening agent
Span 80	1.57	1.57	-	-	-	Emulsifier
Stearic acid	-	-	-	4	4	Emulsifier
Stearyl alcohol	-	-	-	-	-	Emulsifier
2% Carbomer	-	-	-	-	10	Rheology modifier/Thickening agent
Propylene glycol	5	10	5	3	4	Humectant/Levigating agent
Cetomacrogol 1000	1.5	3	5	10	10	Emulsifier
Tween 80	3.43	3.43	-	-	-	Emulsifier
Methyl paraben	1	1	1	0.20	0.20	Preservative
Purified water q.s.	100	100	100	100	100	Vehicle

### 2.7 Evaluation of physicochemical properties of *V. trifolia* cream formulation (HCF)

The physicochemical characteristics of the HCF were evaluated according to pharmaceutically accepted methods and relevant guidelines [22,23]. Briefly, the appearance and homogeneity were evaluated visually based on color, odor, consistency, uniformity, and phase separation. Physical stability of the formulation was further assessed by centrifugation resistance testing. The formulations were identified as oil-in-water (o/w) emulsions based on their miscibility and washability with water. Viscosity was measured using a Brookfield viscometer at 25 °C, while pH was determined using a calibrated pH meter after dispersing the cream in distilled water. Spreadability was evaluated using a glass-slide method by measuring the diameter of cream spread under a 500 g weight. All measurements were performed in triplicate, and results were expressed as mean values.

### 2.8 Stability testing

Stability testing of herbal cream containing *V. trifolia* was carried out using two models of freeze-thaw stability testing and short-term stability testing. For freeze-thaw stability testing, the sample was filled in a bottle and kept in a chamber maintained at 30 ± 2 °C for 24 h, and 4 ± 2 °C for another 24 h, three cycles. At the end of the study, the samples were analyzed for the physical properties, pH, and viscosity. Moreover, their short-term stability was performed for 6 months under temperatures of 6 °C and 4 °C. Every month, the sample was observed for its physical and chemical characteristics [23].

### 2.9 Phytochemical screening of herbal cream formulation 5 (HCF5)

#### 2.9.1 Total phenolic contents

Total phenolic content of HCF5 was assessed by the Folin-Ciocalteu assay according to a previous report with slightly adapted [24]. Briefly, HCF5 at a concentration of 0.125 mg/ml was prepared, and then an aliquot of the sample (100 μl) was mixed with 250 μl of Folin-Ciocalteu reagent diluted in distilled water. The mixture was thoroughly combined and incubated at room temperature for 1 min. Subsequently, 750 μl of 20% sodium carbonate (Na_2_CO_3_) solution was added, and the solution was mixed well and incubated at room temperature for 2 h. The absorbance was measured at a wavelength of 760 nm using a spectrophotometer plate reader. The experiment was performed in quintuplicate (5 times). The total phenolic content of the samples was calculated using a standard curve prepared from gallic acid and expressed as milligrams of gallic acid equivalents per gram of dry extract (mg GAE/g).

#### 2.9.2 Total flavonoid content

The total flavonoid content was determined using the Aluminium Chloride method, with catechin as the standard according to a previous report [25]. HCF5 was prepared at a concentration of 0.125 mg/ml. An aliquot of the sample (200 μl) was mixed with 75 μl of 5% (w/v) sodium nitrite (NaNO_2_) solution and incubated at room temperature for 5 min. Then, 10% aluminium chloride (AlCl_3_) solution (150 ml) was added. The mixture was carefully combined and incubated at room temperature for 6 min. Afterward, 1 M sodium hydroxide (NaOH) solution (5 μl) was added. The absorbance was measured at 510 nm using a spectrophotometer plate reader. The experiment was repeated in quintuplicate (5 times). Catechin was used as a standard control, and the result was expressed as mg of catechin/g of extract (mg CE/g extract).

#### 2.9.3 Total proanthocyanidin content

Total proanthocyanidin content was determined using a modified method based on a previous report [26]. Briefly, HCF5 was prepared at a concentration of 0.125 mg/ml, and an aliquot of the HCF5 solution (1000 μl) was mixed with 2500 μl of 1% vanillin dissolved in methanol, and 2500 μl of 9 M HCl dissolved in methanol. The mixture was combined and incubated at 30 °C for 20 min. The experiment was repeated in quintuplicate (5 times), and the absorbance was measured at 500 nm using a spectrophotometer plate reader. The total proanthocyanidin content of the samples was calculated using a standard curve prepared from catechin and expressed as milligrams of catechin equivalents per gram of dry extract weight (mg CE/g extract).

### 2.10 Statistical analysis

The determination of phytochemical compounds, *in vitro* biological activity results, and *in vivo* anti-inflammatory and analgesic activities are presented as mean ± standard error of the mean (Mean ± S.E.M.). Differences between experimental groups and the control group were evaluated using one-way ANOVA, followed by multiple comparisons with Dunnett's test. Data on physical properties are also reported as mean ± standard error of the mean (Mean ± S.E.M.). Comparisons between pre- and post-experiment data were performed using paired t-tests, while variations across different time points were analyzed using repeated measures ANOVA. Statistical significance was defined as P < 0.05.

## 3 Results

### *3.1* *In vivo* anti-inflammatory activity of VTE on EPP-induced rat ear edema

The results of VTE on EPP-induced ear edema in rats are shown in Table 2. It demonstrated that rats treated with VTE at doses of 0.5 and 1 mg/ear exhibited a significant reduction in ear swelling compared to the control group at all time points, with inhibition percentage ranging from 47.8% to 71.5%. Administering the VTE extract at a concentration of 2 mg/ear before EPP induction significantly reduced ear swelling 30 min after induction, with this effect continuing up to 120 min after induction compared to the control group. It is particularly noteworthy that topical application of VTE at a dose of 0.5 mg/ear was able to prevent swelling in rats to a level comparable to that achieved with indomethacin at a dose of 2 mg/ear.

**Table 2:** The inflammatory effect of VTE on rat ear swelling induced by EPP at 15, 30, 60, and 120 min after EPP induction.

Group	Dose (mg/ear)	Ear edema (μm)			
		15 min	30 min	60 min	120 min
Control	-	134.93 ± 29.94	130.83 ± 20.25	103.61 ± 11.82	82.33 ± 3.12
Indomethacin	2	61.88 ± 10.71* (54.1%)	65.69 ± 13.14** (49.8%)	56.64 ± 13.34* (45.3%)	44.86 ± 8.18** (45.5%)
VTE	0.5	62.67 ± 10.97* (53.6%)	68.33 ± 5.36** (47.8%)	47.22 ± 5.47** (54.4%)	39.44 ± 8.67*** (52.1%)
	1	49.27 ± 10.08** (63.5%)	44.07 ± 4.24*** (66.3%)	37.88 ± 6.67*** (63.4%)	23.43 ± 6.00*** (71.5%)
	2	89.17 ± 7.38 (33.9%)	76.33 ± 13.39* (41.7%)	39.31 ± 10.56*** (62.1%)	44.58 ± 8.16** (45.9%)

Values are expressed as mean ± S.E.M (n = 6). p-value measured by ANOVA followed by Dunnett's multiple comparisons.

*P < 0.05, **P < 0.01, ***P < 0.001 compared to control.

VTE = *Vitex trifolia* ethanolic extract.

EPP = Ethyl phenylpropiolate.

### 3.2 In vivo anti-inflammatory activity of VTE on carrageenan-induced rat paw edema

The results of this study indicate that rats receiving the extract at a dose of 125 mg/kg significantly prevented paw inflammation at 2 and 4 h after inflammation induction, with inhibition values of 40.9% and 35.7%, respectively, compared to the control group. Increasing the extract dose to 250 mg/kg led to a statistically significant reduction in paw swelling at all tested time points compared to the control group, with inhibition values ranging from 37.2% to 50.8%. However, at a dose of 500 mg/kg, the extract significantly prevented inflammation at 4 h after inflammation induction. In comparison, indomethacin at 10 mg/kg significantly reduced paw swelling in rats at all tested time points compared to the control group (Table 3).

**Table 3:** The inflammatory effect of VTE on rat paw edema induced by carrageenan at 1, 2, 3, and 4 h of the induction.

Group	Dose (mg/kg)	Edema volume (ml)			
		1 h	2 h	3 h	4 h
Control	-	0.15 ± 0.01	0.17 ± 0.01	0.17 ± 0.01	0.22 ± 0.02
Indomethacin	10	0.06 ± 0.01*** (60.4%)	0.08 ± 0.02*** (53.4%)	0.08 ± 0.02*** (54.5%)	0.06 ± 0.01*** (73.6%)
VTE	125	0.11 ± 0.01 (29.5%)	0.10 ± 0.00** (40.9%)	0.14 ± 0.01 (19.6%)	0.14 ± 0.02** (35.7%)
	250	0.10 ± 0.01* (37.2%)	0.08 ± 0.02*** (50.8%)	0.08 ± 0.02** (50.5%)	0.12 ± 0.02** (45.3%)
	500	0.12 ± 0.03 (20.4%)	0.13 ± 0.01 (24.2%)	0.15 ± 0.01 (11.4%)	0.15 ± 0.01* (31.2%)

Values are expressed as mean ± S.E.M (n = 6). p-value measured by ANOVA followed by Dunnett's multiple comparisons.

*P < 0.05, **P < 0.01, ***P < 0.001 compared to control.

VTE = *Vitex trifolia* ethanolic extract.

### 3.3 In vivo analgesic effect of VTE on the acetic acid-induced writhing test

The oral administration of VTE at doses of 125, 250, and 500 mg/kg did not result in a statistically significant difference in the number of writhes compared to the control group. However, mice treated with indomethacin at a dose of 10 mg/kg exhibited a statistically significant reduction in the number of writhes, with an average of 11.00 ± 1.06 writhes and an inhibition percentage of 77.63% (Table 4). Notably, administration of VTE at doses of 125-250 mg/kg demonstrated pain-relieving effects within 10 min, but the inhibitory effect diminished over time, whereas the extract at a dose of 500 mg/kg maintained consistent efficacy in reducing the number of writhes in mice (33.3%-37.8%).

**Table 4:** The analgesic effect of VTE in the acetic acid-induced writhing test at 10, 20, and 30 min after the induction.

Group	Dose (mg/kg)	Number of writhing		
		10 min	20 min	30 min
Control	-	49.17 ± 9.39	63.00 ± 14.74	82.83 ± 19.17
Indomethacin	10	11.00 ± 1.06***(77.6%)	20.83 ± 2.34*(66.9%)	27.83 ± 3.85*(66.4%)
VTE	125	40.33 ± 2.89(18.0%)	76.17 ± 3.24(-20.9%)	96.00 ± 4.34(-15.9%)
	250	30.17 ± 4.56(38.6%)	61.67 ± 8.27(2.1%)	78.83 ± 9.90(4.8%)
	500	32.50 ± 9.01(33.9%)	42.00 ± 12.11(33.3%)	51.50 ± 15.39(37.8%)

Values are expressed as mean ± S.E.M. p-value measured by ANOVA followed by Dunnett's multiple comparisons. *P < 0.05, ***P < 0.001 compared to control.

VTE = *Vitex trifolia* ethanolic extract.

### 3.4 In vivo analgesic effect of VTE on the hot plate test

Table 5 presents the results of VTE on analgesic activity using the hot plate test. The administration of VTE at doses of 125, 250, and 500 mg/kg did not result in a significant difference in latency to respond to heat compared to the control group at any time point. In contrast, mice treated with morphine at a dose of 10 mg/kg showed a significant increase in latency to respond to heat at 30, 60, 90, and 120 min after administration compared to the control group (Table 5).

**Table 5:** The analgesic effect of VTE in the hot plate test at 0, 30, 60, 90, and 120 min after the induction.

Group	Dose	Reaction time (sec)				
	(mg/kg)	0 min	30 min	60 min	90 min	120 min
Control	-	6.51 ± 0.48	5.89 ± 0.30	4.91 ± 0.47	4.88 ± 0.76	4.52 ± 0.52
Morphine	10	6.74 ± 0.45	14.40 ± 1.16***	11.67 ± 1.63***	10.79 ± 0.58***	8.36 ± 0.62***
VTE	125	6.30 ± 0.29	6.01 ± 0.75	5.62 ± 0.37	4.84 ± 0.67	3.94 ± 0.49
	250	6.01 ± 0.24	5.02 ± 0.75	5.67 ± 0.74	5.30 ± 0.69	4.58 ± 0.73
	500	6.29 ± 0.32	5.79 ± 0.62	4.77 ± 0.53	4.75 ± 0.63	5.46 ± 0.45

Values are expressed as mean ± S.E.M (n = 6). p-value measured by ANOVA followed by Dunnett's multiple comparisons.

***P < 0.001 compared to control.

VTE = *Vitex trifolia* ethanolic extract.

### 3.5 Pharmaceutical herbal cream formulation

Five formulations of *V. trifolia* herbal cream formulation (HCF1–HCF5) exhibited a semi-solid consistency, a light green color, and a characteristic odor. There was no phase separation under normal conditions. However, after centrifugation, only HCF4 and HCF5 remained stable. All formulations possessed pH values that ranged from 4.26 to 5.03, within the acceptable range for topical application. Viscosity varied among formulations, with HCF4 being the most viscous (40,065 ± 1974 cP) and HCF1 being the least (8684 ± 703 cP). HCF5 displayed the most favorable characteristics, which are moderate viscosity (16,876 ± 1348 cP), pH 4.44 ± 0.18, and high stability. Therefore, HCF5 was selected for further development (Table 6).

**Table 6:** Physical properties of *V. trifolia* herbal cream (HCF1 – HCF5).

Parameters	Samples				
	HCF1	HCF2	HCF3	HCF4	HCF5
Appearance	Semi-solid	Semi-solid	Semi-solid	Semi-solid	Semi-solid
Color	Light green	Light green	Light green	Light green	Light green
Odor	Characteristic	Characteristic	Characteristic	Characteristic	Characteristic
Consistency	Normal^a^	Normal	Normal	Normal	Normal
Phase separation	No	No	No	No	No
Resistance to centrifugation	PS^b^	PS	PS	NPS^c^	NPS
pH	4.99 ± 0.27	5.03 ± 0.09	4.62 ± 0.05	4.26 ± 0.04	4.44 ± 0.18
Viscosity (cP)	8683 ± 703.44	9587 ± 648.95	12,550 ± 782.86	40,065 ± 1974.42	16,876 ± 1348.24

a Normal = Homogeneous and smooth.

b PS = Phase separation.

c NPS=No phase separation.

### *3.6* *Heating-cooling stability testing of* cream base formulation 5 (CBF5) *and HCF5*

The heating-cooling stability test was performed over six cycles to evaluate the physical properties of HCF5 containing VTE, compared to its CBF5. After the testing, neither sample obviously change in consistency, odor, and general appearance. However, phase separation was observed in both HCF5 and CBF5 after the testing, though no separation occurred upon centrifugation. A significant decrease in pH was observed in both formulations following the heating-cooling cycles (P < 0.05), dropping from 5.00 ± 0.02 to 4.32 ± 0.01 in CBF5 and from 4.99 ± 0.01 to 4.32 ± 0.01 in HCF5. A significant reduction in spreadability and an increase in viscosity (P < 0.05) were observed in both samples. The viscosity of CBF5 increased from 19,061 ± 2723 cP to 44,890 ± 1469 cP, while HCF5 increased from 17,166 ± 366 cP to 36,528 ± 1535 cP. Despite these changes, both formulations had acceptable physical characteristics for topical use. The results indicate that HCF5 is physically stable under accelerated temperature (Table 7).

**Table 7:** Evaluation of physical characteristics and stability of cream base formulation 5 (CBF5) and herbal cream formulation 5 (HCF5) using heating-cooling method.

Parameters	CBF 5		HCF 5	
	Before	After	Before	After
Appearance	Semi-solid	Semi-solid	Semi-solid	Semi-solid
Color	White & glossy	NC^a^	Light green	NC
Odor	Unscented	Unscented	A light scent of herbs	A light scent of herbs
Consistency	Homogeneous and smooth	NC	Homogeneous and smooth	NC
Phase separation	NPS^b^	PS^c^	NPS	PS
Resistance to centrifugation	NPS	NPS	NPS	NPS
Spreadability	3.80 ± 0.07	3.03 ± 0.08*	3.95 ± 0.07	2.94 ± 0.13*
pH	5.00 ± 0.02	4.32 ± 0.01*	4.99 ± 0.01	4.32 ± 0.01*
Viscosity (cP)	19,061 ± 2723	44,890 ± 1469*	17,166 ± 366	36,528 ± 1535*

Values are expressed as mean ± S.E.M. (n = 5). p-value measured by Paired test comparisons. *P < 0.05 compared to before heating-cooling cycle.

a NC=No change.

b NPS=No phase separation.

c PS = Phase separation.

### 3.7 Short term stability testing of CBF5 and HCF5 for 90 days

The short-term stability study was performed over 90 days at 30 °C and 4 °C. Both HCF6 and CBF5 were semi-solid appearance and showed no phase separation throughout the study. Slight color changes were observed in HCF5 at 30 °C, while CBF5 remained unchanged under both conditions. Significant reductions in pH were observed in both samples by Day 30 (P < 0.05). Although all values were within the acceptable range for dermal products. Viscosity increased over time in both CBF5 and HCF5. Notably, HCF5 demonstrated greater viscosity stability at 4 °C compared to 30 °C. Spreadability decreased slightly in both samples over time but remained within an acceptable range. Overall, the presence of VTE in HCF5 did not affect the physical stability of the formulation. HCF5 retained good physical characteristics over the 90-day storage period at both temperatures (Table 8).

**Table 8:** Evaluation of short-term stability of cream base formulation 5 (CBF5) and herbal cream formulation 5 (HCF5) stored at 30 °C and 4 °C for 90 days.

Day	Samples	Appearance	Phase separation	Spreadability	pH	Viscosity (cP)
**At 30°C**						
0	CBF5	Normal^a^	NPS^c^	3.80 ± 0.07	5.00 ± 0.02	19,061 ± 2723.62
	HCF5	Normal^b^	NPS	3.95 ± 0.07	4.99 ± 0.01	17,166 ± 366.08
30	CBF5	NC^d^	NPS	3.75 ± 0.03	4.54 ± 0.01*	25,302 ± 755.19
	HCF5	SC^e^	NPS	3.78 ± 0.05	4.40 ± 0.01*^#^	23,682 ± 736.71*
60	CBF5	NC	NPS	3.81 ± 0.04	4.50 ± 0.01*	22,097 ± 545.89
	HCF5	SC	NPS	3.82 ± 0.06	4.29 ± 0.01*^#^	22,182 ± 344.30*
90	CBF5	NC	NPS	3.80 ± 0.06	4.50 ± 0.01*	25,061 ± 1350.76
	HCF5	SC	NPS	3.77 ± 0.06	4.30 ± 0.02*^#^	27,940 ± 1293.72*
**At 4°C**						
0	CBF5	Normal	NPS	3.80 ± 0.07	5.00 ± 0.02	19,061 ± 2723.62
	HCF5	Normal	NPS	3.95 ± 0.07	4.99 ± 0.01	17,166 ± 366.08
30	CBF5	NC	NPS	3.72 ± 0.03	4.48 ± 0.02*	24,883 ± 2588.48
	HCF5	NC	NPS	3.73 ± 0.04	4.56 ± 0.12	16,916 ± 572.78^#^
60	CBF5	NC	NPS	3.82 ± 0.02	4.45 ± 0.01*	22,711 ± 2811.67
	HCF5	NC	NPS	3.84 ± 0.02	4.39 ± 0.01*^#^	15,438 ± 568.69^#^
90	CBF5	NC	NPS	3.76 ± 0.05	4.44 ± 0.01*	23,366 ± 1096.29
	HCF5	NC	NPS	3.93 ± 0.05	4.38 ± 0.00*^#^	15,356 ± 129.41^#^

Values are expressed as mean ± S.E.M. (n = 5). p-value measured by Repeated measure ANOVA (Greenhouse-Geisser). *P < 0.05 compared to Day 0. #P < 0.05 compared to Base.

a Semi-solid, Homogeneous, White & glossy, and unscented.

b Semi-solid, Homogeneous, Light green, and odor characteristic.

c NPS=No phase separation.

d NC=No change.

e SC = Slightly change in color.

### 3.8 Phytochemical evaluation

The phytochemical stability of HCF5 was assessed for 90 days at two temperatures of 30 °C and 4 °C. At baseline (Day 0), the total phenolic content was 50.05 ± 7.93 mg GAE/g extract at both temperatures. Across the study period, slight increases were observed with no significant difference, reaching 60.99 ± 9.94 mg GAE/g at 30 °C and 59.33 ± 8.93 mg GAE/g at 4 °C by Day 90. Total flavonoid content increased over time, from 16.68 ± 1.09 mg CE/g at Day 0 to 28.10 ± 2.43 mg CE/g (30 °C) and 29.78 ± 3.05 mg CE/g (4 °C) by Day 90. Similarly, total proanthocyanidin contents were stable at 30 °C and showed an increased trend at 4 °C (64.73 ± 7.80 mg CE/g to 69.34 ± 16.20 mg CE/g). Despite minor variations, no significant difference was observed in any of the phytochemical constituents, suggesting that HCF5 maintained its bioactive compound integrity under both storage conditions over the 90 days (Table 9).

**Table 9:** Total phenolics, total flavonoids, and total proanthocyanidins of herbal cream formulation 5 (HCF5) stored at 30 °C and 4 °C for 90 days.

Day	Total phenolic contents (mg GAE/g extract)		Total flavonoid contents (mg CE/g extract)		Total proanthocyanidin contents (mg CE/g extract)	
	30 °C	4 °C	30 °C	4 °C	30 °C	4 °C
0	50.05 ± 7.93	50.05 ± 7.93	16.68 ± 1.09	16.68 ± 1.09	64.73 ± 7.80	64.73 ± 7.80
30	65.49 ± 5.68	61.80 ± 1.75	20.59 ± 9.19	20.93 ± 2.66	71.85 ± 4.41	85.21 ± 3.51
60	52.51 ± 15.97	50.06 ± 9.03	24.98 ± 4.15	27.03 ± 7.09	62.79 ± 6.93	74.98 ± 16.29
90	60.99 ± 9.94	59.33 ± 8.93	28.10 ± 2.43	29.78 ± 3.05	45.72 ± 8.50	69.34 ± 16.20

Values are expressed as mean ± S.E.M. (n = 5). p-value measured by Repeated measure ANOVA (Greenhouse-Geisser).

## 4 Discussion

This study was performed based on our previous findings, which demonstrated notable antioxidant, antibacterial, and cell migration-promoting properties, suggesting its potential for topical applications [4]. This current study provides scientific evidence supporting the traditional use of VTE, exhibiting the anti-inflammatory activity *in vivo* and the successful development as a stable topical herbal cream formulation. The *in vivo* pharmacological results, together with the formulation and stability data, provided a comprehensive ethnopharmacological validation of *V. trifolia*, bridging traditional knowledge with modern scientific application.

This current study exhibited that VTE possessed strong anti-inflammatory activity in EPP-induced ear edema and carrageenan-induced paw edema models in rats, which was comparable to the standard anti-inflammatory drug. These results suggested that VTE modulated both early- and late-phase inflammation. Both EPP and carrageenan induce immediate irritation characterized by vasodilation, increased blood flow, vascular permeability, and leukocyte infiltration, leading to fluid accumulation and edema [27,28]. The response involves initial mediator release (e.g., histamine, serotonin, bradykinin), followed by prostaglandins, nitric oxide (NO), and pro-inflammatory cytokines such as IL-1β, IL-6, and TNF-α [27,28]. The observed anti-inflammatory effects of VTE between 15 and 240 min after the induction related with the inhibition of both phases. Consistently, our previous work showed that VTE consisted of high levels of phenolic and flavonoid compounds, contributing to its anti-inflammatory and antioxidant activity [4]. Additionally, previous *in vitro* studies supported that an aqueous extract of *V. trifolia* could inhibit NO and pro-inflammatory cytokines, including IL-1, IL-6, IL-10, and iNOS in LPS-induced inflammation in RAW 264.7 cells [[29], [30], [31]]. Similarly, Annamalai and Thangam (2022) reported that methanolic extracts of *V. trifolia* at doses of 100 and 200 mg/kg significantly attenuated carrageenan-induced inflammation and cytokine levels via NF-κB pathway suppression [32]. Taken together, these findings confirmed the hypothesis that VTE exerts anti-inflammatory effects by inhibiting key inflammatory mediators and signaling pathways.

VTE exhibited weak antinociceptive effects in either peripheral (the acetic acid-induced writhing test) and central (the hot plate assay) pain models. Based on the results of writhing test, the effects of VTE remained statistically comparable to the control group (P > 0.05), and there was no significant increase in latency time. These findings suggested that VTE did not sufficiently relieve pain through both peripheral and central mechanisms. In contrast, a previous report revealed that repeated administration of ethanolic *V. trifolia* extract could reduce the writhing response and increased latency time [33]. This may be attributed to the difference in dosage and administration regimen. Our protocol involved a single dose administered 30 min before the testing, while their study involved three-day pre-treatment. It suggested that the analgesic effect of VTE may require cumulative dose or a longer onset period. Furthermore, according to Thai traditional medicine, the topical use of *V. trifolia* for pain relief is typically integrated with Thai massage, where the mechanical stimulation may contribute significantly to the overall analgesic effect. In this current experiment, the extract was topically applied without other interventions. Therefore, it may not fully reflect the therapeutic context in which this plant is traditionally used. Therefore, further studies are needed to clarify these mechanisms and to investigate the potential analgesic effects of VTE with different administration protocols.

VTE extract was developed into a topical herbal cream formulation (HCF5), which exhibited favorable physical characteristics, including suitable viscosity, spreadability, and pH properties. The formulation was designed as an oil-in-water emulsion (O/W), a dosage form widely preferred for topical applications because of its non-greasy texture, good homogeneity, ease of application, and high patient acceptability. In O/W emulsions, oil droplets are dispersed within a continuous aqueous phase, which facilitates easy washing and improves the sensory characteristics of the formulation. In addition, O/W systems generally provide good spreadability and uniform distribution over the skin surface [23]. Despite phase separation occurring in the heating-cooling test, both HCF5 and CBF5 were restored to a homogeneous characteristic after centrifugation, indicating the physical flexibility and strength of the formula. This response aligns with previous studies demonstrating that temporary instability during heating-cooling test does not necessarily predict the long-term instability if the product can recover upon mechanical reconstitution [[34], [35], [36], [37]]. Moreover, the stability assessment under real-time storage conditions (at 4 °C and 30 °C) over a period of 90 days revealed that HCF5 retained the structural and functional properties with only minor changes in pH and viscosity. Although fluctuations in viscosity were observed during the 90-day period, the herbal cream can be considered physically stable, as the variations remained within an acceptable range. In addition, they were not accompanied by signs of physical instability, such as phase separation, color, and odor. Minor changes in viscosity are commonly reported in semisolid formulations and may result from temperature-dependent rearrangement of the internal structure of the emulsion system during storage [20,38]. According to pharmaceutical stability guidelines, a formulation may be considered stable if there are no significant changes affecting product performance, appearance, or usability [22,39]. Despite viscosity fluctuations, the overall stability profile of the cream remained acceptable. Notably, the formulation stored at 30 °C exhibited superior stability compared to that stored at 4 °C, as evidenced by fewer fluctuations in physical characteristics over time. This finding may be explained by the reduced crystallization or phase disruption at moderate ambient temperatures, a phenomenon commonly reported in emulsion-based creams [[40], [41], [42]]. Such results are particularly relevant for herbal product commercialization in tropical climates, where cold-chain storage may not be feasible. Phytochemical analysis further supported the chemical stability of the formulation. Total phenolics, total flavonoids, and total proanthocyanidins did not show a significant decline during 90 days of storage at both temperatures, consistent with a previous study [42]). These bioactive compounds are well-documented for their antioxidant and anti-inflammatory activities and are considered major contributors to the therapeutic efficacy [4]. Their sustained presence not only supports continued pharmacological activity but also enhances product shelf-life and standardization, which are essential for regulatory approval and consumer confidence in herbal products. Furthermore, the successful development of VTE into a stable topical cream extended the practical applicability of the extract from experiment to formulation science. This finding complements our previous work, which demonstrated the antibacterial activity of VTE against common skin pathogens and the ability to promote cell migration [4]. By integrating anti-inflammatory efficacy with formulation stability, this study provided a holistic ethnopharmacological validation of *V. trifolia*, reinforcing the potential as a standardized topical therapeutic agent for inflammatory skin disorders.

Although this study demonstrated the anti-inflammatory potential of VTE and its applicability in a topical herbal formulation, several limitations should be acknowledged. First, no significant analgesic effects were observed, which may be due to the mechanisms of action not involving central or peripheral nociceptive pathways, or due to limitations in the experimental design. Moreover, the difference in administration routes between morphine and VTE in the hot plate assay may have introduced pharmacokinetic variability, including differences in absorption and onset of action, which could have influenced the comparative antinociceptive outcomes. Secondly, only one concentration (0.4% w/w) of the extract was used in the cream formulation without dose-ranging comparisons. Additionally, the formulation stability assessment relied on basic physical and colorimetric evaluations (e.g., pH, viscosity, total polyphenols), lacking more specific analytical techniques such as HPLC as well as UPLC-MS/MS to confirm qualitative and quantitative compositional stability. Furthermore, no cytotoxicity or skin irritation studies, as well as mechanistic evaluation at the cellular level in RAW264.7 macrophage models, were conducted on the final formulation. Therefore, future studies should address these limitations to support clinical translation of this herbal product.

## 5 Conclusion

This study provides scientific evidence supporting the traditional use of *V. trifolia* for the treatment of inflammatory conditions. VTE exhibited significant anti-inflammatory activity in both topical and systemic animal models, with effects comparable to standard anti-inflammatory agents. Although the extract did not exhibit significant antinociceptive effects in the tested models, which remained comparable to the control group, this outcome may reflect the limitations of a single-dose administration protocol or the sensitivity of the testing methods and does not preclude potential analgesic effects under alternative dosing regimens. In addition, the successful development of a stable HCF5 demonstrated the possibility for dermal application. The cream displayed acceptable physical characteristics and phytochemical stability under both accelerated and real-time storage conditions. These findings supported the potential of *V. trifolia* as a promising candidate for development into a standardized herbal topical product. Further studies exploring inflammatory mechanism, chronic administration, combination therapies, and clinical validation are warranted to advance its ethnopharmacological relevance into therapeutic application.

## Author contribution

WP: Investigation, Data curation, Formal analysis, Writing – original draft, Writing - reviewing & editing.

JC: Conceptualization, Data curation, Formal analysis, Methodology, Project administration, Supervision, Writing – original draft, Writing - reviewing & editing, Funding acquisition.

NK: Methodology, Investigation, Writing – original draft, Writing - reviewing & editing.

KM: Investigation.

JN: Writing – original draft, Writing - reviewing & editing.

## Ethics statement

All procedures involving animals were conducted in accordance with the guidelines of the Prince of Songkla University Animal Ethics Committee. The animal ethics application was submitted on 11 November 2022 and approved on 1 December 2022, under approval number (Ethic Number: MHESI 68014/2083, Ref.AR103/2022).

## Data availability

Data will be made available on request.

## Declaration of generative AI in scientific writing

None.

## Declaration of competing interest

The authors declare no conflict of interest.
